# Maillard reaction products in plant-based dairy alternatives and their release during simulated gastrointestinal digestion

**DOI:** 10.1016/j.crfs.2025.100994

**Published:** 2025-01-30

**Authors:** Kira Bieck, Franziska Ebert, Tilman Grune, Jana Raupbach

**Affiliations:** a Institute of Nutritional Science, Department of Food Chemistry, University of Potsdam, 14469, Potsdam, Germany; b Department of Molecular Toxicology, German Institute of Human Nutrition Potsdam-Rehbruecke (DIfE), 14558, Nuthetal, Germany; c Institute of Food Chemistry, Technische Universität Braunschweig, 38106, Braunschweig, Germany

**Keywords:** CML, CEL, Digestion, Maillard reaction, MG-H1, Plant-based food

## Abstract

Plant-based food products are becoming increasingly popular among consumers. The chemical composition and the processing of plant-based products presumably fuel the Maillard reaction, but the abundance of Maillard reaction products in plant-based food products is rarely investigated. In this study, the concentration of N-ε-carboxymethyllysine (CML), N-ε-carboxyethyllysine (CEL) and methylglyoxal-hydroimidazolone (MG-H1) was analyzed with UPLC-MS/MS in six plant-based dairy alternatives. Total amounts of free and protein-bound glycation compounds ranged from 0.03 to 0.31 mg/100 g food for CML, 0.04–1.28 mg/100 g food for CEL and 0.69–2.84 mg/100 g food for MG-H1. Free glycation compounds were abundant in yogurt and cheese, but not milk alternatives. During simulated gastrointestinal digestion, CML and MG-H1 were released either as modified amino acid or in peptide-bound form, respectively. CEL was released to a significantly lesser extent in peptide-bound form. For CML, de novo formation of up to 400 % during digestion was observed. The results showed that Maillard reaction products are quantitatively important process-induced compounds in plant-based food products which are available after digestion.

## Highlights

• Maillard reaction products were analyzed in plant-based dairy alternatives.
• Plant-based dairy alternatives contain higher amounts than animal-derived products.
• Simulated digestion releases Maillard reaction products from protein backbone.
• Additional Maillard reaction products are formed during digestion of food products.
• Protein source, sugar content and food processing impact intestinal release.

## Graphical abstract

**Figure:**
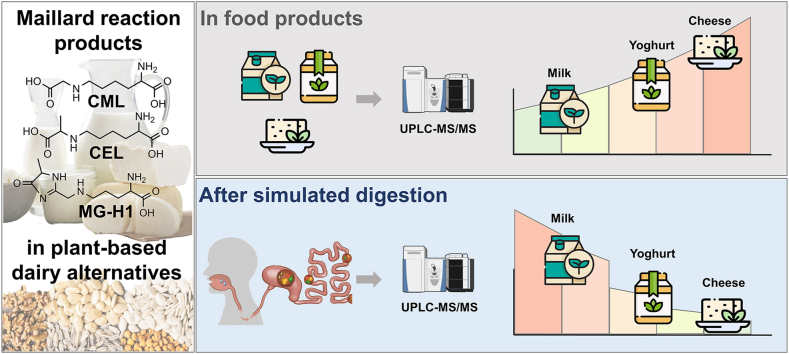

## 1 Introduction

The demand for plant-based food products has been increasing in recent years, due to the growing awareness of the environmental and health benefits of a plant-based diet (Tantamango-Bartley et al., 2013; Barnard et al., 2006; Hedenus et al., 2014; Hallström et al., 2015). A variety of commercial plant-based products try to mimic the taste and experience of eating meat or drinking milk while being marketed as a healthier and more sustainable alternative to animal-derived products. The products are industrially produced from largely processed plant-based ingredients and thus are often defined as ultra-processed food (UPF). Their main purpose is to create convenient, non-perishable food products that are ready-to-eat or heat. This leads to the production of frozen, canned and instant food items (Monteiro et al., 2018). Despite the rather negative perception of food processing, it fulfills various purposes during the production of plant-based products. Mechanical and thermal processing of plant-based products aims to denature trypsin inhibitors, kill pathogens and warrant microbiological safety and improve their technofunctional properties (Gilani et al., 2012; Maganinho et al., 2024). When foods are processed at high temperatures, chemical reactions between proteins, amino acids and reducing sugars can occur in course of the Maillard reaction. The Maillard reaction, also known as glycation, describes the non-enzymatic reaction of reducing sugars with amino groups of proteins and amino acids (Hodge, 1953). In proteins, mainly the side chains of lysine and arginine are modified and a plethora of compounds, so-called Maillard reaction products (MRPs), are formed. The first stable intermediates are Amadori products, such as the condensation product of lysine and glucose, N-ε-fructoselysine (FL). Degradation and consequent reactions of these early glycation products lead to the formation of 1,2-dicarbonyl compounds, such as methylglyoxal and glyoxal, which can further react with proteins and amino compounds to generate so-called advanced glycation endproducts (AGEs). The class of AGEs comprises a large group of structurally diverse compounds. If lysine reacts with 1,2-dicarbonyl compounds or monosaccharides, lysine-derived AGEs, namely N-ε-carboxymethyllysine (CML) and N-ε-carboxyethyllysine (CEL) are formed besides others. The reaction of arginine results, among other products, in the formation of methylglyoxal-derived hydroimidazolone 1 (MG-H1). Due to the modification of essential amino acids such as lysine, the Maillard reaction impacts the nutritional quality of proteins. The health impact of dietary MRPs was extensively discussed recently by Hellwig et al. (2024) (Hellwig et al., 2024). The authors concluded that there is at present no convincing evidence for a causal association between dietary intake of glycation compounds and adverse health effects, but also pointed out that reliable occurrence data for improved exposure assessment is often missing.

Plant-based dairy alternatives are typically supplemented with sweeteners to overcome known nutritional and sensory limitations (Tangyu et al., 2019). Compared to cow's milk, which mainly contains the disaccharide lactose, plant-based products often contain monosaccharides such as glucose or fructose (Milkovska‐Stamenova et al., 2019). The impact of the differing chemical composition of plant-based products compared to their animal-derived counterparts on the formation of Maillard reaction products is unclear. Only limited quantitative data on the abundance of MRPs in plant-based food products is available. For milk it was shown, that ultra-high-temperature-processed cow milk contains up to 8.3 mg CML/100 g protein (Förster et al., 2005), whereas plant-based soy drinks contain 45–130 mg CML/100 g protein (Bosch et al., 2007; Tang et al., 2023). Protein modifications due to the Maillard reaction can impact the nutritional value of proteins by hindering proteolysis and thus limiting the release of amino acids. The intestinal digestive protease trypsin cleaves peptide bonds at the C-terminal side of lysine and arginine residues. Since these amino acids are especially vulnerable towards glycation, proteins are less efficiently hydrolyzed by proteases. Deng et al. (2017) showed that α-lactalbumin glycated with glucose is less efficiently cleaved by lysine/arginine specific proteases, such as trypsin (Deng et al., 2017). In contrast, hydrolysis by proteases which do not bind to lysine or arginine residues, such as α-chymotrypsin and subtilisin A, are less affected. The authors tested different degrees of glycation without changes in secondary, tertiary and quaternary structure of the protein. For plant-derived proteins, Tang et al. showed that glycation of pea and soy protein with glucose decreases gastric digestibility but does not markedly influence intestinal digestibility, probably due to the presence of several proteases in pancreatin compensating the effect of glycation (Tang et al., 2023). The aim of gastrointestinal digestion is to break down food into smaller, absorbable components. During digestion of Maillard-modified proteins, not only native, nutritious amino acids and peptides, but also glycated amino acids or peptides are released. For a model system of casein glycated with glucose and lactose, it was shown that MG-H1 was more efficiently released from the protein backbone with 42 % than CML with 27 % and CEL with 5 % (van der Lugt et al., 2020).

The chemical composition and the processing of plant-based products presumably fuel the Maillard reaction, which leads to protein modification and altered protein quality. Neither the quantitative relevance nor the release of MRPs from plant-based products during digestion is sufficiently studied so far. Therefore, the aim of this study was to quantitatively analyze MRPs in selected plant-based dairy alternatives. Additionally, the release of Maillard reaction products after simulated static gastrointestinal digestion was studied.

## 2 Materials & methods

### 2.1 Chemicals

Chemicals and enzymes used were of standard analytical grade, and were purchased from Sigma-Aldrich Inc. (St. Louis, MO, USA) unless stated otherwise. Pepsin (P7012), α-amylase (A3176), bile (B8631) and pancreatin (P7545; 8 × USP specifications activity) were all from porcine origin. Analytical standards of MRPs and deuterated internal standards were obtained from Iris Biotech (Marktredwitz, Germany). Liquid chromatography solvents were of mass spectrometry grade and obtained from Merck (Darmstadt, Germany). Perfluoropentanoic acid (NFPA) was purchased from Alfa Aesar (Haverhill, USA).

### 2.2 Food products

A set of six food samples was purchased at a supermarket located in Potsdam, Germany. A list of the food products, their plant source and nutritional parameters according to the manufacturers as well as the protein content after analysis by the Kjehldal method are shown in Table 1.

**Table 1:** Plant-based food products.

Food product	Plant source	Sugar content (g/100 g)	Protein content (g/100 g) according to product label	Protein content (g/100 g ± SD) according to analysis
milk alternative	Soy	2.5	3	2.52 ± 0.19
milk alternative with vanilla flavoring	Soy	6.7	3	2.9 ± 0.02
milk alternative	Pea	0	2.4	1.85 ± 0.04
yoghurt alternative	Soy	2.2	4	3.96 ± 0.09
cream cheese alternative	Almond	0.9	6.6	7.16 ± 0.12
feta cheese alternative	Almond	1.0	5	5.54 ± 0.11

### 2.3 Simulated gastrointestinal digestion

*In vitro* simulated digestion was performed according to the INFOGEST protocol (Brodkorb et al., 2019; Minekus et al., 2014). The enzyme activities were measured before the digestion experiment as described previously (Brodkorb et al., 2019). In brief, an aliquot of 5 mL of the liquid food samples or 5 g of the solid food samples was mixed with 5 mL of simulated salivary fluid (pH 7, 37 °C) and incubated at 37 °C for 2 min. Then, 10 mL of simulated gastric juice (pH 3, 37 °C) containing pepsin (2000 U/mL of digesta) were added and incubated for 120 min. Subsequently, 20 mL of simulated intestinal juice (pH 7, 37 °C) containing pancreatin (100 U trypsin activity/mL of digesta) and bile (10 mmol/L of total digesta) were added and incubated for 120 min. The digestion protocol was performed at 37 °C under constant gentle mixing in a water bath. Simulated digestion was stopped by the addition of 9 mL TCA (20% w/v in water) to 1 mL of the digested samples. The samples were cooled to −20 °C for 30 min, centrifuged (10 min, 10000g, 4 °C) and the supernatant was stored at −20 °C for subsequent analysis. A blank sample containing water was included in the analysis.

### 2.4 Analysis of free Maillard reaction products

The term “free MRPs” refers to the lysine (CML, CEL) or arginine modification (MG-H1) without any protein- or peptide-backbone present.

For analysis of food samples, aliquots of the yoghurt and cheese alternatives were dissolved in 0.5 mL of water to obtain a protein concentration of 1 mg/mL. Of the milk alternatives, 0.5 mL were used in undiluted form. For protein precipitation, 1 mL of ACN was added to the samples. After placing the samples on ice for 1 h, samples were centrifuged (10 min, 9400×*g*, 4 °C). For subsequent analysis, 90 μL of supernatant were used.

For analysis of digestion samples, 90 μL of the supernatant after TCA protein precipitation were used. The 90 μL aliquots of supernatants from food or digestion samples were mixed with 10 μL internal standard mixture (d4-CML 24.0 μM, d4-CEL 22.5 μM, d3-MG-H1 21.6 μM) and subsequently quantified via UPLC-MS/MS.

### 2.5 Analysis of Maillard reaction products bound to proteins or peptides

**Fig. 1:**
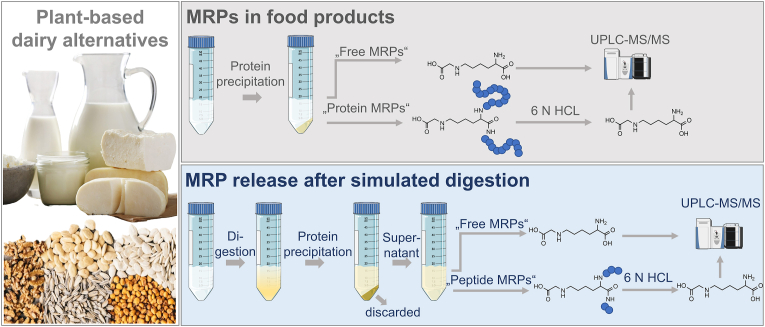
Work flow for the analysis of free and protein-bound MRPs in food samples, as well as free and peptide-bound MRPs in digestion samples.

In food samples, proteins were acid precipitated, followed by acid hydrolysis and subsequent analysis of MRPs. Therefore, bound MRPs in food samples are referred to as “protein-bound”. In digestion samples, proteins were acid precipitated and discarded. The supernatant, containing low-molecular-weight MRPs, was subjected to acid hydrolysis prior to UPLC-MS/MS analysis. Therefore, bound MRPs in digestion samples are referred to as “peptide-bound”. The work flow for the analysis of free and protein-bound MRPs in food samples, as well as free and peptide-bound MRPs in digestion samples is shown in Fig. 1.

Analysis in food samples was performed following Scheijen et al. (2016) (Scheijen et al., 2016) with slight modifications. Briefly, food samples were dissolved as described previously and 25 μL of the samples were mixed with 250 μL borate buffer (0.4 N, pH 10.2) and 250 μL sodium borohydride (1M in 0.1 M NaOH) and incubated at room temperature for 2 h. Subsequently, 1 mL TCA (20% w/v in purified water) was added and samples were centrifuged (10 min, 9400×*g*, 4 °C). The supernatant was removed and the residue was washed with 1 mL TCA (20% w/v in purified water) and centrifuged (10 min, 9400×*g*, 4 °C). The supernatant was removed and 10 μL of the internal standard mix (d4-CML 24.0 μM, d4-CEL 22.5 μM, d3-MG-H1 21.6 μM) and 1 mL HCl (6M) were added to the residue. The solution was incubated at 110 °C for 23 h and subsequently dried with a vacuum concentrator. The dry residue was dissolved in 100 μL aqueous UPLC solvent (10 mM NFPA in water), centrifuged (10 min, 9400×*g*, 4 °C) and 80 μL were transferred into a HPLC vial with insert and analyzed by UPLC-MS/MS.

For analysis of digestion samples, 1000 μL of the supernatant after TCA protein precipitation was dried with a vacuum concentrator. To the dry residue, 250 μL borate buffer (0.4 N, pH 10.2) and 250 μL sodium borohydride (1M in 0.1 M NaOH) were added and the samples were incubated at room temperature for 2 h. Samples were dried with a vacuum concentrator and 10 μL of internal standard mix (d4-CML 24.0 μM, d4-CEL 22.5 μM, d3-MG-H1 21.6 μM) and 1 mL HCl (6M) were added to the dry residue. The solution was incubated at 110 °C for 23 h and subsequently dried with a vacuum concentrator. The dry residue was dissolved in 100 μL aqueous UPLC solvent (10 mM NFPA in water), centrifuged (10 min, 9400×*g*, 4 °C) and 80 μL were transferred into a HPLC vial with insert and analyzed by UPLC-MS/MS.

### 2.6 UPLC-MS/MS

UPLC-MS/MS analysis was performed with an Acquity Ultra Performance LC system coupled to a Waters TQ-XS mass spectrometer (both Waters Corporation, Milford, MA, USA) using ESI positive multiple reaction monitoring mode (MRM). For chromatographic separation, an Acquity UPLC BEH C18 column, (1.7 μm, 2.1 mm × 50 mm, Waters Corporation) at a column temperature of 40 °C was used. For chromatographic separation, a flow rate of 0.6 mL/min, an injection volume of 10 μL and a gradient elution of solvent A (10 mM NFPA in water) and solvent B (10 mM NFPA in ACN) was chosen (gradient: 0 min, 1% B; 1 min, 1% B; 6 min, 40% B; 8 min, 80% B; 9 min, 80% B; 9.1 min, 1% B; 12 min, 1% B). For electrospray ionization, a capillary voltage of 2.7 kV with a desolvation gas flow of 650 L/h at 350 °C was applied. Analytes were measured in positive MRM mode with the following transitions and optimized collision energies (CE) and cone voltages (CV). CML: 204.9 → 84.2 (q, CV 24, CE 18 V), 204.9 → 130.2 (Q, CV 24, CE 12 V), d4-CML: 209.2 → 88.1 (q, CV 24, CE 20 V), 209.2 → 134.1 (Q, CV 24, CE 12 V), CEL: 219.1 → 84.1 (q, CV 24, CE 18 V), 219.1 → 130.1 (Q, CV 24, CE 12 V), d4-CEL: 223.2 →88.1 (q, CV 24, CE 20 V), 223.2 →134.2 (q, CV 24, CE 12 V), MG-H1: 229.2 → 70.1 (q, CV 26, CE 22 V), 229.2 → 113.6 (Q, CV 26, CE 14 V), d3-MG-H1: 232.2 → 73.1 (q, CV 26, CE 22 V), 232.2 → 116.6 (Q, CV 26, CE 14 V). Transitions used for quantification are labeled with q and transitions used for the confirmation of the presence of the analyte are labeled with Q. External calibration for CML (0.12–12.24 μM), CEL (0.11–11.45 μM) and MG-H1 (0.11–10.95 μM) was performed. Data were acquired and evaluated with the MassLynx Software (Waters, version 4.1).

### 2.7 Determination of protein content

Protein quantification of the food samples was performed with the Kjeldahl method. Briefly, 0.5–1.5 g of the food sample, catalyst (Na_2_SO_4_ 47.7%, K_2_SO_4_ 47.7%, TiO_2_ 2.8%, CuSO_4_ 1.8%, mercury- and selenium-free; Merck, Darmstadt, Germany), boiling beads, and 10 mL sulfuric acid were heated to 400 °C for about 2 h until the solution turned clear. Additionally, a blank sample and urea as reference were treated the same way. After the reaction was completed, ammonia was captured in 20 mL boric acid (2 %) with steam distillation. The solution containing ammonium ions, proportional to the protein content in the food sample was titrated using 0.05 M H_2_SO_4_. The following equation was used with the conversion factor of 6.25 to calculate the protein content:

$$\%\;protein=\frac{\left[V{\left(0.05\;M\;H_{2}SO_{4}\right)}_{sample}-V{\left(0.05\;M\;H_{2}SO_{4}\right)}_{blank}\right]*1.4*100*6.25}{m_{sample}}$$

### 2.8 Statistical analysis

Graphing and statistical analysis was performed with Graph Pad Prism version 8.0.1 (San Diego, USA). All data are presented as mean values of n = 3 ± SD.

## 3 Results & discussion

### 3.1 Protein content

A recovery rate of 96.7 % was determined with urea for the Kjeldahl method. The protein contents of the food products were quantified and the results are shown in Table 1. The milk alternatives had slightly lower protein concentrations than stated by the manufacturers. For further calculations, the protein content analyzed with the Kjeldahl method were used.

### 3.2 Quantification of Maillard reaction products in plant-based dairy products

**Fig. 2:**
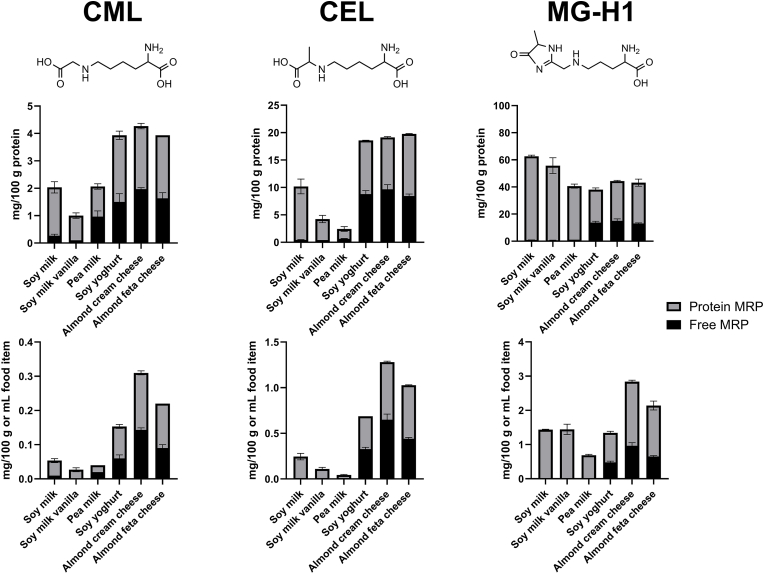
**Concentration of free and protein-bound CML, CEL and MG-H1 in plant-based dairy alternatives.** Structures of free MRPs and concentration of free (black) and protein-bound (grey) CML, CEL and MG-H1 related to protein content (upper row) or related to the mass or volume of the food samples (lower row). The total bar represents the total concentration of the respective MRP in the food sample. Mean values of n = 3 ± SD.

The concentrations of free and protein-bound MRPs in the food samples were determined via UPLC-MS/MS. In food samples, the term “free” MRPs refers to the lysine (CML, CEL) or arginine modification (MG-H1) without any protein- or peptide-backbone present. The term “protein-bound” MRPs includes all amino acid modifications at a protein backbone which were released thereof after hydrolysis with 6 N HCl. The mean concentrations of free and protein-bound CML, CEL and MG-H1 are shown in Fig. 2, either related to the protein content in the upper row or related to the mass (yoghurt and cheese alternatives) or volume (for milk alternatives) in the lower row.

Free CML ranged from 0.002 mg/100 mL for soymilk vanilla to 0.141 mg/100 g for cream cheese. Protein-bound CML ranged from 0.021 mg/100 mL for pea milk to 0.165 mg/100 g for cream cheese. Regarding CEL, the free form ranged from 0.01 mg/100 mL for soymilk vanilla to 0.65 mg/100 g for cream cheese. CEL in its protein-bound form ranged from 0.03 mg/100 mL for pea milk to 0.63 mg/100 g for cream cheese. The lowest concentration of free MG-H1 was quantified in pea milk with 0.02 mg/100 mL, the highest in cream cheese with 0.97 mg/100 g. Protein-bound MG-H1 ranged from 0.67 mg/100 mL in pea milk to 1.86 mg/100 g in almond cream cheese. The highest total concentration of each MRP was quantified in the cream cheese alternative. No correlation of the sugar concentration and the amount of total MRPs was observed. The soy milk with vanilla flavoring has a sugar content of 6.7 g/100 g but contains rather low amounts of CML, CEL and MG-H1. In contrast, the almond cream cheese with a rather low sugar concentration of 0.9 g/100 g, showed the highest concentrations of all three MRPs. The soy milk alternatives as well as the yoghurt alternatives contain sucrose which is a non-reducing carbohydrate and thus does not contribute substantially to the Maillard reaction. Analysis of carbohydrates of the food products and quantification of reducing sugars could provide more information in the future. For all MRPs and food samples, the concentrations of the protein-bound form exceed the free form. The solid samples (yoghurt, cream cheese, feta cheese) contain higher amounts of the free MRPs compared to the liquid samples (soymilk, soymilk vanilla, and pea milk), what also leads to higher levels of total MRPs in yoghurt, cream cheese and feta cheese. The increased levels of free MRPs in these products might be explained by the manufacturing process. Plant-based milk alternatives are commonly produced by blending the respective plant part with water and different additives. The production of plant-based yoghurt or cheese alternatives involves several steps, including fermentation (Kovačević et al., 2024). Glycated proteins might be partially hydrolyzed during fermentation, leading to an increased concentration of free MRPs.

**Fig. 3:**
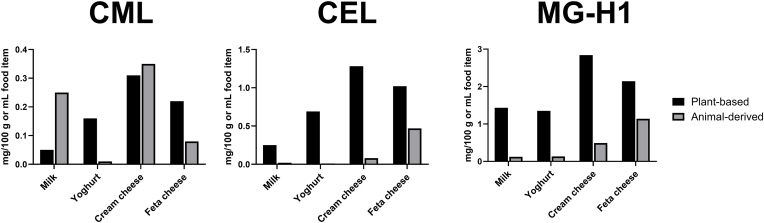
**Comparison of total concentration of MRPs in plant-based and animal-derived products.** Comparison of total concentration of CML, CEL and MG-H1 in plant-based alternative (black bar) and animal-derived product (grey bar). Plant-based products derived from soy (milk, yoghurt) or almond (cheeses), animal-derived products originated from cows (milk, yoghurt, cream cheese) or sheep (feta cheese). Concentrations of animal-derived products are adapted from Scheijen et al. (2017) (Scheijen et al., 2016). Concentrations of plant-based dairy alternatives are mean values of n = 3 measurements.

Comparing the total amounts of MRPs (free and protein-bound), CML is present in the lowest concentration in a range of 0.03–0.31 mg/100 g food item, followed by CEL with 0.04–1.28 mg/100 g food item and MG-H1 ranging from 0.69 to 2.84 mg/100 g food item. Previously, the concentration of CML, CEL and MG-H1 has been quantified in animal-derived dairy products via UPLC-MS/MS (Scheijen et al., 2016). Fig. 3 shows the comparison of MRP concentrations in animal-derived products (data adapted from (Scheijen et al., 2016)) with their respective plant-based alternatives (this study).

For CML, depending on the food product, the concentration is similar, higher or lower in the plant-based product, compared to the animal-derived product. For CEL and MG-H1, the concentration in the plant-based alternatives is higher than in the traditional animal-derived products. Intense processing of plant-based products might explain increased levels of MRPs, but does not help to account for the different abundances of CML versus CEL and MG-H1 in animal- and plant-derived products. CML, CEL and MG-H1 result from reaction of the ε-amino group of lysine or the guanidino group of arginine with the dicarbonyl compounds glyoxal (GO) or methylglyoxal (MGO) (Ahmed et al., 1986, 1997). CML is also formed from the oxidation of the Amadori compound fructosyllysine or the Schiff base intermediate (Nagai et al., 1997; Kasper and Schieberle, 2005). Whereby for CML a formation pathway independent of the presence of 1,2-dicarbonyl compounds exist, the formation of CEL and MG-H1 relies on the presence of these sugar degradation products. The concentration of 1,2-dicarbonyl compounds in plant-based milk alternatives was recently shown to be significantly higher compared to cow-derived UHT milk (Pucci et al., 2024). The increased levels of 1,2-dicarbonyl compounds in plant-based milk alternatives might fuel the formation of CEL and MG-H1 since their formation depends on the presence of the reactive intermediates. Another factor which might further enhance the formation of 1,2-dicarbonyl-compound-derived MRPs, such as CEL and MG-H1, is the lack of substantial amounts of creatine in plant-based products. Creatine is involved in muscle synthesis in humans and animals and therefore is abundantly present in meat and dairy products. Vegetarian diets are nearly absent in creatine compared to non-vegetarian diets (Blancquaert et al., 2018). Creatine reacts with MGO in food and in vivo and forms N-(4-methyl-5-oxo-1-imidazolin-2-yl)sarcosine (MG-HCr), which is renally excreted after dietary consumption. Urinary excretion of MG-HCr is influenced by dietary habits and differences between omnivores, vegetarians and vegans have been observed (Löbner et al., 2015; Treibmann et al., 2019, 2020). In meat and dairy products MGO will partially react with creatine to form MG-HCr and therefore will not be available for the formation of MGO-derived MRPs. This might explain higher concentrations of MGO-derived MRPs, such as CEL and MG-H1, compared to CML in plant-based dairy products.

### 3.3 Release of Maillard reaction products during simulated gastrointestinal digestion

**Fig. 4:**
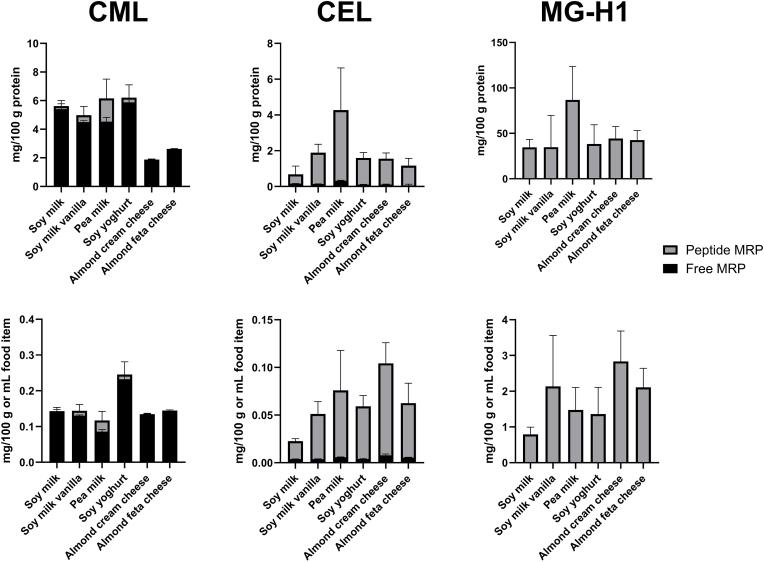
**Concentration of free and peptide-bound CML, CEL and MG-H1 after simulated digestion.** Concentration of free (black) and peptide-bound (grey) CML, CEL and MG-H1 related to protein content of the native food product (upper row) and related to the mass or volume of the food samples (lower row). The total bar represents the total concentration of the respective MRP after digestion. Mean values of n = 3 ± SD.

The concentration of MRPs in the plant-based products does not correspond to the actual concentration of MRPs which is available for resorption after gastrointestinal digestion. To determine the effective concentration of CML, CEL and MG-H1 in the gastrointestinal tract, a simulated digestion of the plant-based dairy products was performed. Subsequently, the undigested fraction was removed with acid precipitation and MRPs were quantified in the supernatant. In digestion samples, the term “free” MRPs refers to the lysine (CML, CEL) or arginine modification (MG-H1) without any protein- or peptide-backbone present. The term “peptide-bound” MRPs includes all amino acid modifications in the supernatant bound to smaller peptides which were released thereof after hydrolysis with 6 N HCl. Fig. 4 shows the mean concentrations and standard deviations of free and peptide-bound MRPs after simulated digestion, either related to the protein content of the native food product (upper row) or related to the mass or volume of the food product (lower row).

After digestion, CML is mainly present in its free form, while CEL and MG-H1 are predominantly or exclusively present in their respective peptide-bound forms. Free MRPs were directly analyzed in the supernatant after TCA precipitation of the digested samples. For analysis of peptide-bound MRPs, a dried aliquot of the supernatant after TCA precipitation was hydrolyzed with hydrochloric acid to liberate MRPs bound to peptides. According to Yvon et al. (1989) (Yvon et al., 1989) who studied the solubility of casein-derived peptides in 12 % TCA, peptides with up to 8 residues remained in solution whereas peptides with more than 30 residues were precipitated. For peptides with 9–29 residues the solubility was dependent on their primary structure and the amount of hydrophobic amino acids, such as tyrosine, phenylalanine or proline. The more hydrophobic amino acids were present, the less soluble the peptides became. In regard to our study, it is not known which peptides of what size are present in the supernatant after TCA precipitation. Based on the assumption that peptides up to 30 residues were present in the supernatant after TCA precipitation, it raises the question whether the analysis of these peptides is of physiological relevance. It is assumed that only di- and tripeptides can cross the epithelial border and enter the circulation (Miner-Williams et al., 2014), and this was also confirmed for glycated dipeptides (Hellwig et al., 2011). However, larger peptides remaining in the digesta can be hydrolyzed by peptidases present on the enterocytic brush border (Miner-Williams et al., 2014) and thus can still contribute to the dietary load of absorbable glycated compounds. Therefore, the analysis of also larger peptides after acid hydrolysis in the supernatant is reasonable, but more conclusive results could be obtained by peptide analysis directly.

**Fig. 5:**
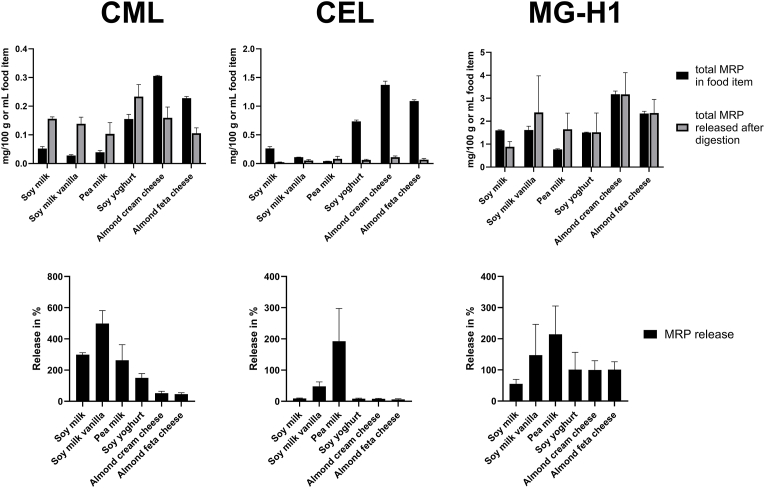
**Release of CML, CEL and MG-H1 during simulated digestion.** Concentration of total CML, CEL and MG-H1 in the food products (black bar) and release of free and peptide-bound MRPs after simulated digestion (grey bar) in the upper row. Release in percent for CML, CEL and MG-H1 in the lower row. Mean values of n = 3 ± SD.

In order to evaluate the release of MRPs during digestion, free and peptide-bound MRPs were summed up and compared to the concentration of MRPs in the food products. Fig. 5 shows the concentration of CML, CEL and MG-H1 in the digesta compared to the concentrations analyzed in the food products prior to digestion.

**Fig. 6:**
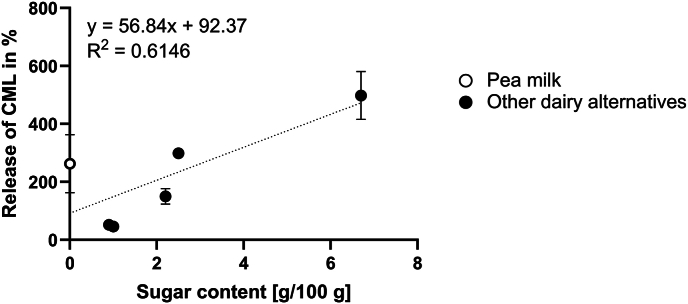
**Correlation of sugar content and the release of CML during simulated digestion.** A positive correlation of the sugar content of plant-based dairy products and the amount of CML released during digestion is shown. Data points represent sugar-free pea milk (○) and all other dairy alternatives (●). Sugar content obtained from the product label, release of CML are mean values of n = 3 measurements.

For CML, a release above 100 % was observed for the milk and yoghurt alternatives and 50–60 % was released from the almond-based cream cheese and feta cheese alternatives. A release above 100 % hints to the fact that more than the amount present in the original food product is recovered and that de novo formation of MRPs occurred. This additional formation is especially abundant for CML showing recovery rates of up to 500 % for the soy milk vanilla sample. Fig. 6 shows the release of CML in dependence to the sugar content of the food products.

Fig. 6 shows a clear trend with a coefficient of determination of 0.6146 towards increased CML formation during gastrointestinal digestion in the presence of higher sugar concentrations in the food samples. According to linear regression, for every gram of sugar in the food products, approximately 60 % of CML which is present in the food sample before digestion is released during digestion. A sugar content of 2 g in the dairy alternatives already leads to a release of CML above 100 % and hence de novo formation during digestion. An increase in the total concentration of MRPs after in vitro digestion has been shown previously (van der Lugt et al., 2020) with recoveries of CML, CEL, and MG-H1 of 200–400 % in the intestinal digests of ginger biscuits and apple juice. Also, the formation of additional MRPs during in vitro digestion of ovalbumin and fructose was demonstrated (Bains et al., 2017). The implications of de novo formation of MRPs in the enteral tract are quite unknown. The quantitative contribution of enterally formed protein modifications to the total amount of protein modifications in the diet is unclear. Enteral modifications of proteins may further impact protein digestibility and should be considered when physiological consequences are investigated. Therefore, further research on enteral MRP formation is needed to fully understand the chemical principles and the potential health implications.

One sample not fitting the trend of increased CML levels in the presence of high sugar contents is the pea milk alternative, which is explicitly advertised as sugar-free. The sugar content was not analyzed in our study but was taken from the nutritional labeling of the products. During simulated digestion, salivary α-amylase was added, which hydrolyzes starch into smaller chains like maltose and dextrins. The starch degradation products might be further hydrolyzed to monosaccharides by pancreatic enzymes, however this remains speculative, since the concentration of reducing sugars was not quantified after digestion. Peas are reported to consist of 60 % carbohydrates, whereby 49 % thereof is starch (Nadathur et al., 2017). The hydrolysis of starch and the release of glucose during simulated digestion of pea milk might lead to de novo formation of CML. The other milk alternatives are based on soybeans, which have a significantly lower carbohydrate content of 15 % (Nadathur et al., 2017). Lower amounts of carbohydrates might limit the liberation of reducing sugars and thus CML de novo formation. However, added sugar, such as present in both soy milk samples, apparently counteracts this limitation.

The plant-based alternatives for cream cheese and feta cheese show an incomplete release of CML during digestion. The concentration of CML after digestion is with 0.13 mg/100 g for cream cheese and 0.14 mg/100 g for feta cheese close to the concentration of free CML quantified before simulated digestion with 0.14 and 0.09 mg/100 g. This might be explained by the lower digestibility of almond-derived proteins compared to soy- and pea-based products (Ahrens et al., 2005).

In contrast to CML, the lysine modification CEL is present mainly in its peptide-bound form and is released between 6 % from feta cheese and 48 % from soymilk vanilla during digestion. When the free CEL concentration prior to digestion and after digestion is compared, only 1–50 % could be recovered. Free CEL might bind to proteins during digestion, precipitate during TCA treatment after digestion and thus withstands analysis. This could be tested by investigating the undigested protein fraction which was acid precipitated and not analyzed in this study. Moreover, free glycated amino acids could be substrates for further glycation reactions, so-called heterogeneous multiple glycation, as recently shown for CML (Hellwig et al., 2022). The twofold glycated compounds would not be detected with the applied transitions during UPLC-MS/MS analysis and thus would be overlooked. The pea-derived milk sample showed a release of 192 % and thus an increase of CEL modifications in peptide-bound form during digestion. De novo formation of CEL in the pea-derived sample could be explained by the breakdown of high amounts of starch in peas and the release of reactive monosaccharides as previously postulated for the formation of CML in pea milk. The analysis of monosaccharides after simulated digestion would be a valuable contribution and should be performed in the future.

The release of MG-H1 was approximately 100 % or higher in all food products. MG-H1 was quantified exclusively in peptide-bound form after simulated digestion. Whether free MG-H1 binds to proteins during digestion and thus evades quantification, could be studied by analyzing the TCA-precipitated protein residue. Following the example of CML, free MG-H1 might also be a target for multiple glycation, leading to yet unknown reaction products. The soy milk vanilla and the pea milk sample showed a 147 % and 214 % release of MG-H1, indicating de novo formation of MG-H1 during digestion. This is line with the data for CML and CEL and could potentially be explained with the high sugar concentration of 6.7 g/100 g of the soymilk vanilla sample and the high starch level in the peas.

In conclusion, this study presents novel insights into the MRP content of plant-based dairy alternatives, as no database regarding these food products exists yet. Higher CEL and MH-H1 concentrations were shown in plant-based dairy alternatives compared to the animal-derived products. These higher concentrations might be explained by the lower concentrations of creatine in plant-based food products, which acts as a scavenger for MGO, a precursor compound for the formation of both CEL and MG-H1. As plant-based and animal-derived food products do not show the same chemical characteristics, further quantification of MRPs in different plant-based alternatives is of scientific interest. During a simulated in vitro digestion, CML and MG-H1 were released completely, whereas CEL was only released in minor concentrations from plant-based dairy alternatives. Additionally, de novo formation of CML during simulated digestion was shown, which correlates with the sugar content of the food sample. Furthermore, this pilot study indicates that food products derived from different plant sources show different behaviors regarding the release of MRPs during in vitro digestion. In this study, soy, almond, and pea protein was analyzed. However, a variety of plant-based alternatives based on other plant proteins, such as oat or lupine already exist. Therefore, the comparison of similar products based on a larger variety of plant proteins could give further insight into the effects of the protein sources on the release of MRPs and protein digestibility in general.

Taken together, this study demonstrates the quantitative relevance of MRPs in plant-based dairy products and their release during gastrointestinal digestion. The results open new perspectives for the investigation of the Maillard reaction in plant-based food products.

## CRediT authorship contribution statement

**Kira Bieck:** Investigation, Formal analysis, Visualization, Writing – original draft. **Franziska Ebert:** Writing – review & editing, Supervision. **Tilman Grune:** Resources, Supervision, Writing – review & editing. **Jana Raupbach:** Conceptualization, Methodology, Supervision, Writing – original draft.

## Declaration of competing interest

The authors declare that they have no known competing financial interests or personal relationships that could have appeared to influence the work reported in this paper.

## Data Availability

Data will be made available on request.
